# Development and Validation of a Long-Term 3D Glioblastoma Cell Culture in Alginate Microfibers as a Novel Bio-Mimicking Model System for Preclinical Drug Testing

**DOI:** 10.3390/brainsci11081025

**Published:** 2021-07-31

**Authors:** Miodrag Dragoj, Jasmina Stojkovska, Tijana Stanković, Jelena Dinić, Ana Podolski-Renić, Bojana Obradović, Milica Pešić

**Affiliations:** 1 Institute for Biological Research “Siniša Stanković”, National Institute of Republic of Serbia, University of Belgrade, 11060 Belgrade, Serbia; miodrag.dragoj@ibiss.bg.ac.rs (M.D.); tijana.andjelkovic@ibiss.bg.ac.rs (T.S.); jelena.dinic@ibiss.bg.ac.rs (J.D.); ana.podolski@ibiss.bg.ac.rs (A.P.-R.); 2 Faculty of Technology and Metallurgy, University of Belgrade, 11000 Belgrade, Serbia; jstojkovska@tmf.bg.ac.rs (J.S.); bojana@tmf.bg.ac.rs (B.O.); 3 Innovation Center of the Faculty of Technology and Metallurgy, University of Belgrade, 11000 Belgrade, Serbia

**Keywords:** glioblastoma, 3D cell culture, alginate hydrogel, temozolomide, drug resistance

## Abstract

Background: Various three-dimensional (3D) glioblastoma cell culture models have a limited duration of viability. Our aim was to develop a long-term 3D glioblastoma model, which is necessary for reliable drug response studies. Methods: Human U87 glioblastoma cells were cultured in alginate microfibers for 28 days. Cell growth, viability, morphology, and aggregation in 3D culture were monitored by fluorescent and confocal microscopy upon calcein-AM/propidium iodide (CAM/PI) staining every seven days. The glioblastoma 3D model was validated using temozolomide (TMZ) treatments 3 days in a row with a recovery period. Cell viability by MTT and resistance-related gene expression (*MGMT* and *ABCB1*) by qPCR were assessed after 28 days. The same TMZ treatment schedule was applied in 2D U87 cell culture for comparison purposes. Results: Within a long-term 3D model system in alginate fibers, U87 cells remained viable for up to 28 days. On day 7, cells formed visible aggregates oriented to the microfiber periphery. TMZ treatment reduced cell growth but increased drug resistance-related gene expression. The latter effect was more pronounced in 3D compared to 2D cell culture. Conclusion: Herein, we described a long-term glioblastoma 3D model system that could be particularly helpful for drug testing and treatment optimization.

## 1. Introduction

Glioblastoma is one of the most common and aggressive types of central nervous system (CNS) tumors, with a 5-year survival rate of only 5.8% [1]. First-line care for the most primary CNS tumors is surgical resection. The standard clinical protocol for glioblastoma after surgical resection is radiation with concomitant and adjuvant chemotherapy [2]. This protocol is known as a Stupp protocol and based on results of phase 3 clinical trial published in 2005, which show that concomitant temozolomide (TMZ) therapy added to radiotherapy followed by 6 cycles of maintenance TMZ (5 days TMZ treatments followed by 23 days of recovery) improves patient survival by only 2.5 months compared to radiotherapy alone [3].

Despite great efforts to introduce more effective therapies, multiple therapies and therapeutic approaches for glioblastoma have been tested, but they all failed to prolong patients’ survival [3,4]. The obvious stagnation in the implementation of new drugs and treatment strategies for glioblastoma is partly due to the lack of appropriate glioblastoma models for testing new drugs that faithfully replicate the clinical scenario.

Currently, traditional two-dimensional (2D) monolayer cell culture is the most commonly used as an in vitro model for understanding tumor cell biology, mechanisms of cancer progression, and the drug discovery process [5]. However, 2D models, due to their simplicity, fail to correctly imitate the 3D environment and the important environmental cues, which often leads to unsuccessful and contradictory results [6,7]. Recently, different 3D bio-mimicking human glioblastoma cell culture models have been advanced to more faithfully reconstitute glioblastoma complexity and better mimic its response to therapy [8]. Spheroids are widely used glioblastoma 3D models, which enable high-throughput testing of various therapeutic options with higher reproducibility at an affordable price [9]. Next to the spheroid, various scaffold-based 3D glioblastoma models were developed with the intention to mimic interactions between cancer cells and the extracellular matrix (ECM) [9,10]. Among them, alginate is one of the most commonly used polymers for microencapsulation and the development of scaffold-based 3D cell cultures. Alginate hydrogels offer several advantageous properties (i) they are affordable and available in large quantities; (ii) biocompatible without causing any toxicity; (iii) can be easily dissolved to release the cells for further analyses; (iv) allow quick diffusion of gases and nutrients through the hydrogel; (v) chemically stable; and (vi) transparent allowing optical monitoring [11].

Regardless of the type, 3D glioblastoma models are generally more resistant to drug treatment than 2D cell cultures showing more similarity with responses observed in glioblastoma patients [12,13,14]. However, all the proposed models did not sustain viable cell cultures for longer periods up to one month to provide at least one clinically relevant treatment cycle, which is important for anti-glioblastoma drug screening studies. Dai et al. cultured 3D bioprinted glioma stem cell model for three weeks, and during that period, the cells did not lose their inherent characteristics. However, in this model TMZ was administrated 24 h after bioprinting, while treatment lasted only 48 h [12]. Other authors compared 2D vs. 3D U87 model during 10 days in respect to growth and phenotype characteristics as well as resistance to chemotherapeutics, including TMZ [13]. Wang et al. characterized a co-culture model of patient-derived GBM cells and mouse brain endothelial cells showing the proliferative and adhesive properties of co-cultured cells at day 1 and day 7 [14].

Therefore, our aim was to establish a long-term 3D culture of human U87 glioblastoma cells in alginate microfibers and validate its suitability for drug testing. Specifically, we optimized and examined the cell immobilization procedure and the influence of the needle diameter and cell density on cell viability. Then, we used TMZ treatments to evaluate the novel glioblastoma 3D model by assessment of cell viability and resistance-related gene expressions.

## 2. Materials and Methods

### 2.1. Cell Line and Cell Culture

The human glioblastoma U87 cell line was purchased from American Type Culture Collection (ATCC, Manassas, VA, USA). The U87 cell line was grown in Minimum Essential Medium (Sigma-Aldrich, Darmstadt, Germany) supplemented with 10% fetal bovine serum (Sigma-Aldrich, Darmstadt, Germany), 2 mM glutamine (Sigma-Aldrich, Darmstadt, Germany), 5000 U/mL penicillin, 5 mg/mL streptomycin (GibcoTM, Thermo Fisher Scientific, Waltham, MA, USA), and 1% MEM Non-essential Amino Acid Solution (Sigma-Aldrich, Darmstadt, Germany). Cells were cultivated at 37 °C in a humidified 5% CO_2_ atmosphere, and passage was performed twice a week after reaching 80–90% confluence using 0.25% trypsin/EDTA (Sigma-Aldrich, Darmstadt, Germany).

### 2.2. Production of Alginate Microfibers with Immobilized Cells

Alginate microfibers with cells were produced by extrusion as described earlier [15]. Briefly, Na-alginate powder (AppliChem, Darmstadt, Germany) was dissolved in deionized water at a concentration of 2% *w*/*w* and sterilized by boiling for 30 min under magnetic stirring. Then, the U87 cell suspension was mixed with the alginate solution to obtain the final concentrations of 1.5% *w*/*v* alginate. In order to determine the most optimal cell concentration, 3 different cell concentrations (1 × 10^6^ cells/mL, 2 × 10^6^ cells/mL, and 4 × 10^6^ cells/mL) were used in the production of alginate microfibers. The cell suspension in alginate was manually extruded through a blunt edge stainless steel needle (22, 25, and 28 gauge-G) immersed in the gelling bath (3% *w*/*v* Ca(NO_3_)_2_ × 4H_2_O). Due to the exchange of Na^+^ with Ca^2+^, the liquid stream solidified in the gelling bath, thus forming insoluble microfibers. The microfibers were left in the bath for 15 min in order to complete gelling and then were washed in the medium.

After cell immobilization, samples (0.5 g of alginate fibers) were distributed into a T25 flask (Sarstedt, Nümbrecht, Germany) and cultured for 28 days without passage in a 13 mL of MEM medium at 37 °C in a humidified 5% CO_2_ atmosphere. The half of the medium was changed twice a week. One group of cells immobilized in alginate microfibers was treated with 100 µM TMZ (Temozolomide, Sigma-Aldrich, Darmstadt, Germany). The treatment started on day 7 and lasted for 3 consecutive days.

### 2.3. Viability Study

**Figure 1:**
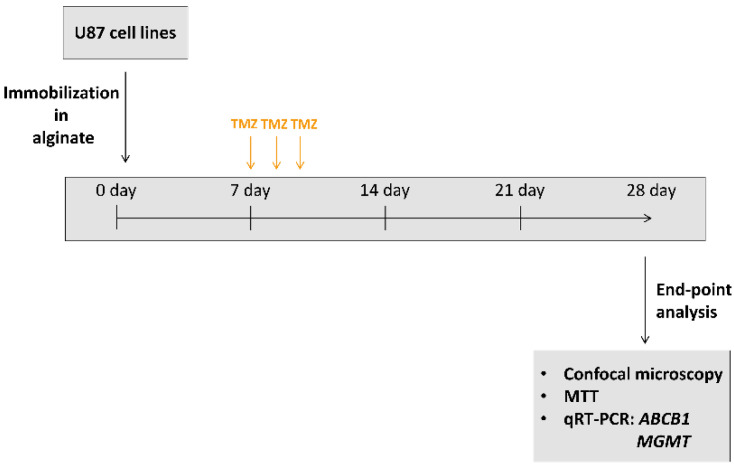
Treatment schedule in long-term 2D and 3D cell cultures.

The U87 cells immobilized in alginate microfibers were stained using CalceinAM (CAM)/propidium iodide (PI) as LIVE/DEAD staining to evaluate the viability of cells directly after alginate microfibers production (day 1), over the incubation time (day 7, 14, 21 and 28) and to examine the efficacy of TMZ treatment. The treatment schedule applied to both 2D and 3D glioblastoma cell culture is presented in Figure 1. Alginate microfibers with cells were incubated for 45 min at 37 °C in a medium supplemented with CAM in a final concentration of 4 μM while PI was added to a final concentration of 5 μM. The U87 cells were imaged using a Leica TCS SP5 II Basic confocal laser-scanning microscope (Leica Microsystems CMS GmbH, Wetzlar, Germany). Live (green) and dead (red) cells were visualized in the alginate microfibers at every *z*-axis encompassing the alginate microfiber. Projections of the z-stack images and quantitative estimation of the cell mass were analyzed using ImageJ software (cell volume in alginate fibers as an indirect measure of cell number). Briefly, the images were first converted to “binary”, and the Otsu method was used to set a threshold range. Within the region of interest (ROI), the area was measured on each slice individually; area measurements were then summed, and this sum was multiplied by the depth of each slice. The result represents the volume of threshold pixels in the ROI of an image stack.

### 2.4. MTT Assay

Cell viability was assessed by the MTT assay (Sigma-Aldrich, Darmstadt, Germany), which was based on the reduction of 3-(4,5-dimethyl-2-thizolyl)-2,5-diphenyl-2H-tetrazolium bromide into formazan dye by active mitochondria in living cells. After 28 days, samples (0.1 g of alginate microfibers with immobilized U87 cells) from the control and treated groups were distributed into 24-well tissue culture plates (Sarstedt, Nümbrecht, Germany) and 500 μL MTT solution (1 mg/mL) was added to each well. The incubation time was 4 h at 37 °C. After removing MTT, the formazan product was dissolved in 500 μL dimethyl sulfoxide, and its absorbance was measured at 540 nm using an automatic microplate reader (Multiskan Sky, Thermo Scientific, Waltham, MA, USA).

### 2.5. RNA Extraction and RT-PCR

After 28 days of incubation, alginate microfibers with U87 cells were dissolved and cells were released. To release cells, alginate microfibers were exposed to dissolving solution containing 0.5 M EDTA, 0.01% *w*/*v* HEPES and 1 × phosphate-buffered saline (Sigma, St. Louis, MO, USA) for 10 min at 37 °C. Total RNA was extracted from the control and treated U87 cells. The same protocol was used for U87 cells cultured in 2D. The extractions were carried out using Trizol^®^ reagent (Invitrogen Life Technologies, Waltham, MA, USA) according to the manufacturer’s instructions. The RNAs were quantified by spectrophotometric analysis, and their quality was determined by agarose gel electrophoresis. cDNA was synthesized using 2 μg total RNA and a high-capacity cDNA reverse transcription kit (Applied Biosystems, Foster City, CA, USA) according to the manufacturer’s instructions.

### 2.6. Quantitative Real-Time PCR

Quantitative real-time PCR (qRT-PCR) was used to assess differences in *MGMT* and *ABCB1* gene expression levels. The primer sequences were as follows: 5′-GCA ATT AGC AGC CCT GGC A-3′ (sense) and 5′-CAC TCT GTG GCA CGG GAT-3′ (antisense) for *MGMT*, 5′-CCC ATC ATT GCA ATA GCA GG-3′ (sense), 5′-GTT CAA ACT TCT GCT CCT GA-3′ (antisense) for *ABCB1*, and 5′-TGG ACA TCC GCAAAG ACC TGT AC-3′ (sense) and 5′-TCA GGA GGA GCA ATG ATC TTG A-3′ (antisense) for *ACTB*. PCR reactions were performed using a Maxima SYBR Green/ROX qPCR Master Mix (Thermo Fisher Scientific, Waltham, MA, USA) on a QuantStudio 3 Real-Time PCR system (Thermo Fisher Scientific, Waltham, MA, USA) according to the manufacturer’s recommendations. The reaction was carried out using 100 ng cDNA in conjunction with specific primers for each gene and *ACTB* as an internal control for normalization. All experiments were performed in triplicate, and relative gene expression levels were analyzed by the 2−ddCt method [16].

### 2.7. Assessment of U87 Cell Proliferation in Real-Time (2D Cell Culture)

Continuous cell proliferation of U87 glioblastoma cells untreated and treated with 100 µM TMZ after 7 days from initial seeding was analyzed using the xCELLigence Real Time Cell analyzer (ACEA Biosciences Inc., San Diego, CA, USA), which enabled label-free real-time cell growth follow-up by measuring impedance-based signals across a series of gold electrodes.

Using E-plates, 50 μL of complete medium was added to each well, and the electrodes were allowed to stabilize for 30 min. The plates were then moved into the xCELLigence Real Time Cell analyzer to set a baseline without cells. Afterward, the cells were seeded on an E-plate at 4000 cells in a 200 μL medium per well. Cells on the electrodes were monitored by reading and recording the cell impedance every 30 min through 2400 sweeps.

### 2.8. Statistical Analysis

Statistical analyses were performed using the statistical package R (R version 4.0.2, Copyright (C) 2020 The R Foundation for Statistical Computing). The statistical analyses were performed by Student *t*-test and Two-way ANOVA and Tukey’s method post hoc test were used to explore differences between multiple groups. Difference was considered to be statistically significant if *p* < 0.05.

## 3. Results

### 3.1. Selection of the Optimal Alginate Microfibers Size for Long-Term 3D Culturing

**Figure 2:**
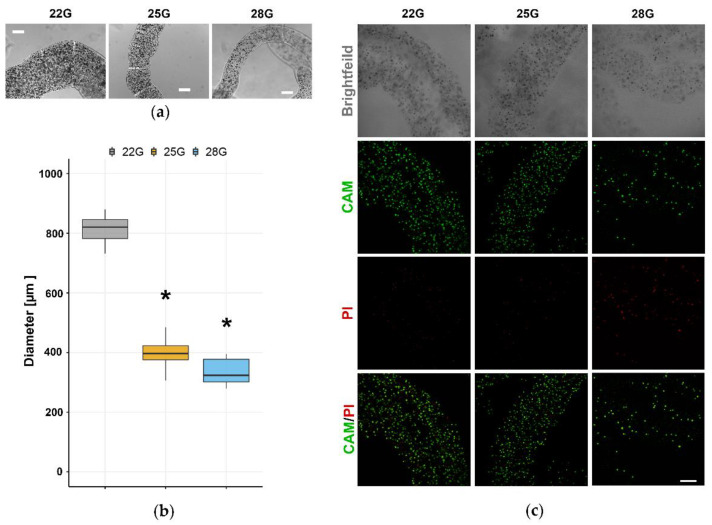
Selection of the optimal alginate microfibers size for long-term 3D culturing. U87 cells were immobilized in alginate microfibers by using different blunt edge stainless steel needle sizes (22, 25, and 28 G). (**a**) Brightfield images were used to determine the microfiber diameter and the results are presented as a (**b**) box-plot. (**c**) LIVE/DEAD monitoring of U87 cells in alginate microfibers stained with CAM/PI was performed by confocal microscopy. Images show live cells labeled with CAM (green) and dead cells labeled with PI (red). Scale bar = 200 µm. All experiments were performed at least three times (*n* ≥ 3). * indicates *p* < 0.05 statistical difference compared to the 22 G blunt edge stainless steel needle.

Ca-alginate hydrogel scaffold was used to create a long-term 3D glioblastoma cell culture in the form of microfibers. Uniform alginate microfibers with immobilized U87 cells were produced by simple extrusion of the alginate solution with cells directly into the gelling bath containing Ca^2+^ (0.18 M). To obtain the fiber diameter for the optimal diffusion of nutrients, three different blunt edge stainless steel needles (22, 25, and 28 G) were used. By using a 22 G needle to extrude a suspension of U87 cells and alginate (4 million U87 cells/1 mL of 2% *w*/*v* alginate), 820 ± 40 μm diameter was obtained (Figure 2a,b). This large diameter did not support the long-term culturing of cells due to the inability of nutrients to diffuse through the fibers and reach all the cells. Significantly smaller diameters of alginate microfibers were obtained using 25 G and 28 G needles. Specifically, a diameter of 400 ± 40 μm was obtained using 25 G, while 28 G needle produced a diameter of 340 ± 40 μm (Figure 2a,b). However, the extrusion process using a 28 G needle affected the morphology of the U87 cells changing their size and granularity (Figure 2a,b).

To investigate the influence of the extrusion process on cell viability, we used live/dead assay based on CAM/PI staining of immobilized U87 cells in alginate microfibers. After extrusion, the majority of cells remained viable in alginate microfibers produced with needles of larger diameter (22 G and 25 G, Figure 2c). However, the extrusion process with 28 G needle and the accompanying shear stress extremely impaired U87 cell viability and reduced the cell density due to the fact that many cells were destroyed and thus not observable in the alginate microfibers (Figure 2c). Based on the abovementioned results, a 25 G needle was used for the process of U87 cell immobilization in alginate microfibers in all subsequent experiments.

### 3.2. Selection of the Optimal Cell Density for Long-Term 3D Culturing

**Figure 3:**
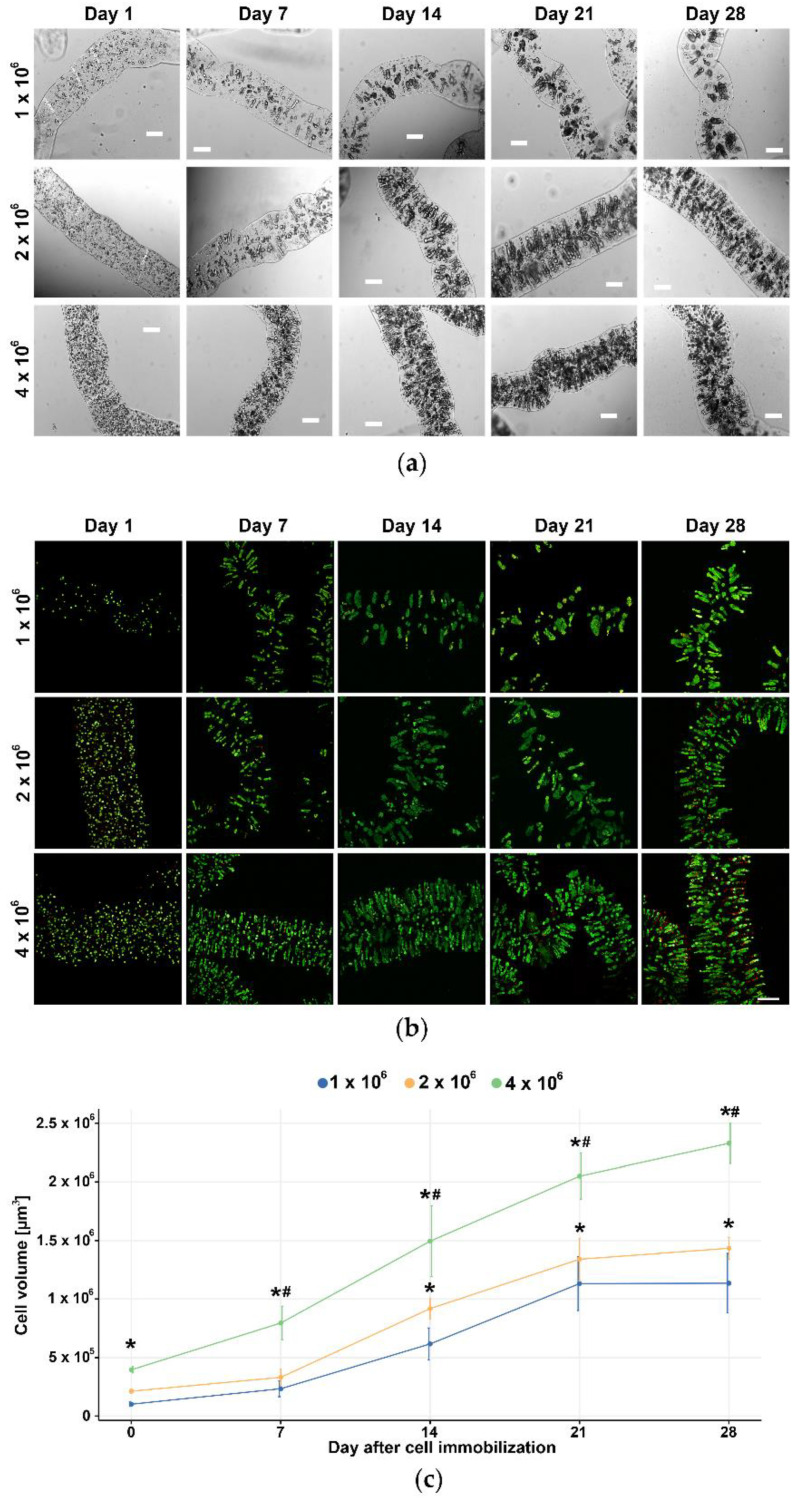
Selection of the optimal cell density for long-term 3D culturing. (**a**) Brightfield light microscopy and (**b**) confocal microscopy of three different U87 cell inoculation densities (1 × 10^6^ cells, 2 × 10^6^ cells, and 4 × 10^6^ cells per 1 mL of 2% *w*/*w* alginate) in long-term 3D cell culture at different time points (day 1, 7, 14, 21, and 28). Merged channels show live cells labeled with CAM (green) and dead cells labeled with PI (red). (**c**) ImageJ software was used to calculate the cell volume in alginate fibers as an indirect measure of cell number. Scale bar = 200 µm. All values are expressed as mean ± SD (*n* ≥ 3). * indicates *p* < 0.05 statistical difference compared to the 1 × 10^6^ cells inoculation densities. # indicates *p* < 0.05 statistical difference compared to the 2 × 10^6^ cells inoculation densities.

Three different inoculation cell densities (1 × 10^6^ cells, 2 × 10^6^ cells, and 4 × 10^6^ cells per 1 mL of 2% *w*/*v* alginate) were monitored under confocal microscopes upon double staining with CAM/PI to assess cell growth, viability, morphology, and aggregate formation. The U87 cells at all three densities grew and stayed viable for 28 days in alginate microfibers (Figure 3a). On the 7th day post-immobilization, U87 cells formed visible aggregates oriented to the microfiber periphery perpendicular to the axis of the fiber, towards the source of oxygen and nutrients. During 28 days in the long-term 3D culture, the aggregates increased in size and cells remained viable even in large aggregates (Figure 3b). The analysis of cell growth demonstrated an increase over time at all tested densities, while the cell death rate was low (Figure 3c). Although all three U87 cell densities were acceptable for further validation of the long-term 3D cell culture, we selected 4 × 10^6^ cells to study the effects of TMZ treatment in better-mimicking conditions with reasonably condensed cells closer to the in vivo glioblastoma cell density.

### 3.3. Validation of Long-Term 3D Glioblastoma Cell Culture with TMZ Treatments

The effects of TMZ treatment on U87 cell viability and resistance-related gene expression (*MGMT* and *ABCB1*) were used to validate and compare our long-term 3D with 2D glioblastoma cell culture. Treatments with 100 μM TMZ started on day 7 and lasted for three consecutive days in both model systems (Figure 1). Then, the medium with TMZ was replaced with the fresh one and the cells were left for recovery during next 18 days.

**Figure 4:**
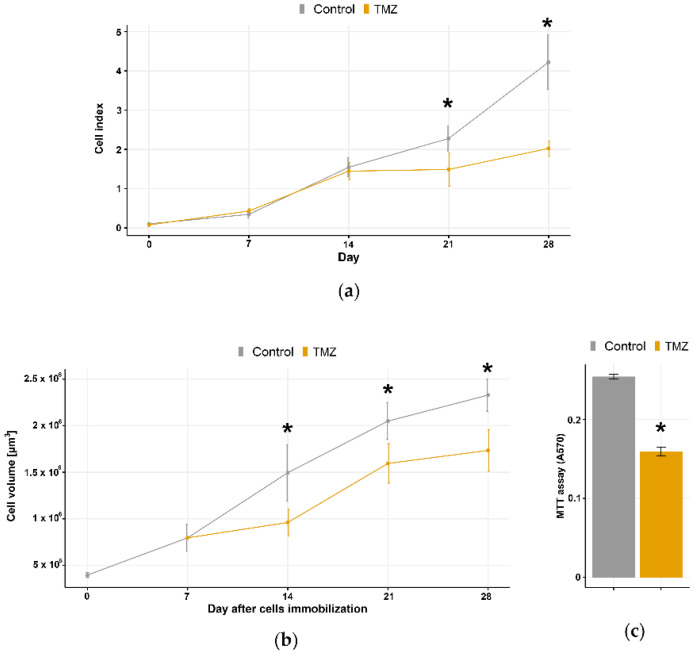
Anti-proliferative effect of TMZ treatments in 2D and 3D long-term cell cultures. Continuous monitoring of U87 glioblastoma cells after the TMZ (**a**) was assessed by xCELLigence real-time cell counter, which enables label-free real-time cell growth follow-up by measuring impedance-based signals expressed as Cell Index across a series of gold electrodes. (**b**) Effects of TMZ treatment on U87 cells in long-term 3D cell culture were assessed by LIVE/DEAD assay, and ImageJ software was used to calculate the Cell volume [µm^3^] in alginate microfibers as an indirect measure of cell number. Viability of the U87 cells in long-term 3D cell culture after the TMZ treatments was assessed by the MTT assay. (**c**) The absorbance at the wavelength 570 nm is presented. All values are expressed as mean ± SD (*n* ≥ 3). * indicates *p* < 0.05 statistical difference compared to the corresponding control cells.

TMZ showed an anti-proliferative effect in both 2D model (assessed by real-time cell counter) and 3D model (calculated by cell volume in alginate microfibers). The successive TMZ treatment suppressed the U87 cell growth more efficiently in 2D model (Figure 4a,b). However, the difference between untreated and treated U87 3D cell culture in the number of viable cells (≈25%) assessed by MTT at the final time point was significant (Figure 4c).

**Figure 5:**
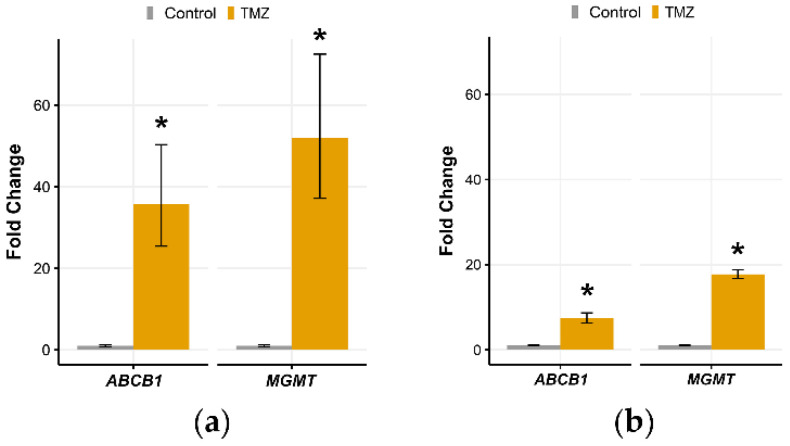
Increased expression of resistance-related genes after TMZ treatments in 2D and 3D long-term cell cultures. Quantitative real-time PCR analyses of *ABCB1* and *MGMT* gene in long-term (**a**) 3D and (**b**) 2D U87 cell culture assessed after TMZ treatments and a recovery period. The mRNA from U87 cells was collected at the final time point (day 28). The expression of target genes was normalized to the *ACTB* gene as internal control and is presented as a relative value compared to the corresponding untreated control. Each sample was tested in triplicate. All results represent mean values ± SD (*n* ≥ 3). * indicates *p* < 0.05 statistical difference compared to the corresponding control cells.

TMZ treatment significantly increased the expression of resistance-related genes *MGMT* and *ABCB1* in 3D and 2D models (Figure 5a,b). The expression study was performed using mRNA from U87 cells collected at the final time point-28th day. The increase in *MGMT* and *ABCB1* expression was more pronounced in the 3D model (Figure 5a).

## 4. Discussion

3D cell culture represents an in vitro model that can bypass most of the shortcomings of 2D cell culture, such as the inability to reproduce 3D cancer architecture. Recently, several 3D bio-mimicking human glioblastoma cell culture models have been developed. These models that more faithfully reconstitute glioblastoma complexity were successfully used for drug screening [8]. However, until now, there were no 3D glioblastoma cell cultures able to mimic the time frame and duration of treatment cycles typically administered to glioblastoma patients.

In this study, we established a 28-days lasting viable U87 glioblastoma 3D cell culture that could be used to mimic one treatment cycle for testing drug effects in a clinically relevant time span. Other authors reported 21-days lasting viable 3D bioprinted glioma stem cell culture, but they did not try to mimic a TMZ treatment cycle [12]. By showing the applicability for the evaluation of TMZ effects in a time course relevant for the clinical practice, our model could be valuable for novel anti-glioblastoma agents testing.

We used alginate hydrogel for the immobilization of U87 cells in the form of microfibers. Among all scaffolds, alginate is one of the most suitable for cell immobilization due to its biocompatibility, permeability, and capability to encapsulate cells in isotonic solutions under physiological pH and temperature. Alginate, with high water absorption capacity, enables the transport of oxygen, nutrients, and growth factors [11,17]. Microencapsulation of the cells in alginate hydrogel by electrostatic extrusion is commonly used to form spheroid cell culture [18,19]. In our model, we avoided using electrostatic extrusion due to its effect on cell viability. Therefore, we immobilized U87 cells in alginate microfibers by using only physical extrusion through the needle.

The extrusion process had several optimization steps in order to obtain sustainable, long-term 3D cell culture. Based on previously published data, we decided to mix 2% *w*/*v* sodium alginate solution with the cell suspension to obtain a final concentration of 1.5% *w*/*v* alginate [20]. Further, the transport of oxygen and nutrients by diffusion through alginate hydrogels is limited to 200 µm [21]. Therefore the diameter of alginate microfibers that would allow delivery of nutrients to immobilized cells should not exceed 500 µm. For the extrusion of the alginate with U87 cells suspension, we tested blunt edge stainless steel needles of different diameters (22 G, 25 G, and 28 G). Although extrusion with 25 G and 28 G needles produced alginate microfibers with preferable diameters (400 µm and 340 µm, respectively), the viability of immobilized cells with the use of a 28 G needle was compromised and cellular morphologically altered. This was caused by increased shear stress due to the extrusion through the needle of a small diameter [22].

Another important factor in the optimization study is the initial number of cells used to obtain 3D cultures. Previous studies with alginate scaffolds reported initial inoculation numbers from 1 × 10^5^ to 1 × 10^7^ cells, depending on the experimental purposes [20,23,24,25,26]. In our model system, we used three different inoculation densities of U87 cells (1 × 10^6^, 2 × 10^6,^ and 4 × 10^6^ cells/mL alginate), and all of them gave rise to the 3D culture that preserved proliferative potential during 28 days. After 7 days, U87 cells started to form visible aggregates oriented to the alginate microfiber periphery towards the source of oxygen and nutrients. The number and size of cell aggregates constantly increased over time due to unrestricted proliferation and a low cell death rate. To obtain better mimicking conditions with more condensed cells as it is in vivo, we selected the highest cell density for further validation of 3D culture with TMZ treatments. Aiming to evaluate TMZ response in a viable setting, our model could not completely replicate the growth and invasion of glioblastoma cells in vivo. Namely, the majority of cells within the alginate microfibers were supplied with enough nutrients, and they did not suffer from the lack of oxygen that drives the glioblastoma growth and spreading from the necrotic core [9].

To verify the applicative potential of established long-term 3D glioblastoma cell culture for drug testing purposes in the clinically relevant time span, we used consecutive TMZ treatments starting from the 7th day of culturing. The starting day of treatments was selected according to the observed formation of U87 cell aggregates 7 days post-immobilization. Treatment with 100 µM TMZ was repeated for three consecutive days followed by 18 days of recovery in a fresh medium. The effects of TMZ on cell viability and resistance-related gene expression were analyzed on the 28th day. We used 100 µM TMZ as IC50 concentration for U87 reported in several studies [27,28]. When compared to 2D glioblastoma cell culture, our 3D culture was more resilient to TMZ thus resembling the response to TMZ in glioblastoma patients [8].

Our results are in accordance with previous findings that resistance to TMZ in 3D glioblastoma cultures increases due to the elevated *MGMT* expression levels [13,29,30]. Thus, we were able to detect the *MGMT* expression levels in U87 cells after 28 days of cultivation in alginate microfibers and show the effects of prolonged successive TMZ treatment. The other abovementioned models presented the TMZ effects only after a single treatment, while the exposure to TMZ lasted 3, 7, or 14 days. In addition, the ATP-binding cassette (ABC) transporter superfamily plays an important role in glioblastoma resistance to chemotherapy [31,32]. Among them, P-glycoprotein (ABCB1) is the most studied, and its role in the blood-brain-barrier is well-established [33]. Its increased expression and role in glioblastoma resistance have been reported [13,30,33,34] as well as its direct interaction with TMZ [35]. Our results demonstrated that besides *MGMT*, *ABCB1* expression also increases in 3D glioblastoma cell culture more profoundly than in 2D. To the best of our knowledge, this is the first time that increased expression of *MGMT* and *ABCB1* upon TMZ treatment followed by a recovery period is observed in a long-term 3D glioblastoma cell culture.

Recently, a sophisticated long-term 3D glioblastoma spheroid cell culture has been reported [36]. This platform enabled 3D volumetric monitoring up to 70 days of patient-derived glioblastoma cells for drug testing purposes. This system included complex methodology of collagen and gelatin bio-printing, co-culturing with endothelial cells, and second-generation mesoscopic fluorescence molecular tomography, which cannot be afforded by many cell culture laboratories. On the other hand, our aim was to establish a model that can be used and evaluated by other research groups offering reproducibility of results, but we are aware of the limitations that should be overcome in the future (i) lack of the continuous flow and exchange of nutrients and metabolic products; (ii) lack of the evidence on the patient-derived cells; (iii) lack of the real-time monitoring of cell behavior; and (iv) lack of the complex microenvironment mimicking in alginate microfibers.

## 5. Conclusions

Herein we presented a novel long-term 3D glioblastoma model with the potential for application in preclinical studies. The advantages of our model include (i) simplicity of establishing in an average cell culture facility; (ii) reproducibility of results; and (iii) ability to test drugs in a clinically relevant time span (at least one cycle of treatment followed by a recovery period).

The proposed 3D culture model system has already shown a significant potential for practical use, resembling some of the important features of glioblastoma cells in vivo, and further system improvements are envisioned to lead to the development of rapid, reliable, and personalized procedures for anticancer drug testing.

## Data Availability

The data presented in this study are available on request from the corresponding author. The data are not publicly available due to lack of institutional online database.

## References

[1] Ostrom Q.T., Cote D.J., Ascha M., Kruchko C., Barnholtz-Sloan J.S. (2018). Adult Glioma Incidence and Survival by Race or Ethnicity in the United States From 2000 to 2014. JAMA Oncol..

[2] Tan A.C., Ashley D.M., Lopez G.Y., Malinzak M., Friedman H.S., Khasraw M. (2020). Management of glioblastoma: State of the art and future directions. CA Cancer J. Clin..

[3] Stupp R., Mason W.P., van den Bent M.J., Weller M., Fisher B., Taphoorn M.J., Belanger K., Brandes A.A., Marosi C., Bogdahn U. (2005). Radiotherapy plus concomitant and adjuvant temozolomide for glioblastoma. N. Engl. J. Med..

[4] Rajaratnam V., Islam M.M., Yang M., Slaby R., Ramirez H.M., Mirza S.P. (2020). Glioblastoma: Pathogenesis and Current Status of Chemotherapy and Other Novel Treatments. Cancers.

[5] Kapalczynska M., Kolenda T., Przybyla W., Zajaczkowska M., Teresiak A., Filas V., Ibbs M., Blizniak R., Luczewski L., Lamperska K. (2018). 2D and 3D cell cultures—A comparison of different types of cancer cell cultures. Arch. Med. Sci. AMS.

[6] Weigelt B., Ghajar C.M., Bissell M.J. (2014). The need for complex 3D culture models to unravel novel pathways and identify accurate biomarkers in breast cancer. Adv. Drug Deliv. Rev..

[7] Lv D., Hu Z., Lu L., Lu H., Xu X. (2017). Three-dimensional cell culture: A powerful tool in tumor research and drug discovery. Oncol. Lett..

[8] Stankovic T., Randelovic T., Dragoj M., Stojkovic Buric S., Fernandez L., Ochoa I., Perez-Garcia V.M., Pesic M. (2021). In vitro biomimetic models for glioblastoma-a promising tool for drug response studies. Drug Resist. Updates Rev. Comment. Antimicrob. Anticancer. Chemother..

[9] Souberan A., Tchoghandjian A. (2020). Practical Review on Preclinical Human 3D Glioblastoma Models: Advances and Challenges for Clinical Translation. Cancers.

[10] Gomez-Roman N., Stevenson K., Gilmour L., Hamilton G., Chalmers A.J. (2017). A novel 3D human glioblastoma cell culture system for modeling drug and radiation responses. Neuro-Oncology.

[11] Lee K.Y., Mooney D.J. (2012). Alginate: Properties and biomedical applications. Prog. Polym. Sci..

[12] Dai X., Ma C., Lan Q., Xu T. (2016). 3D bioprinted glioma stem cells for brain tumor model and applications of drug susceptibility. Biofabrication.

[13] Lv D., Yu S.C., Ping Y.F., Wu H., Zhao X., Zhang H., Cui Y., Chen B., Zhang X., Dai J. (2016). A three-dimensional collagen scaffold cell culture system for screening anti-glioma therapeutics. Oncotarget.

[14] Wang C., Li J., Sinha S., Peterson A., Grant G.A., Yang F. (2019). Mimicking brain tumor-vasculature microanatomical architecture via co-culture of brain tumor and endothelial cells in 3D hydrogels. Biomaterials.

[15] Stojkovska J., Djurdjevic Z., Jancic I., Bufan B., Milenkovic M., Jankovic R., Miskovic-Stankovic V., Obradovic B. (2018). Comparative in vivo evaluation of novel formulations based on alginate and silver nanoparticles for wound treatments. J. Biomater. Appl..

[16] Livak K.J., Schmittgen T.D. (2001). Analysis of relative gene expression data using real-time quantitative PCR and the 2(-Delta Delta C(T)) Method. Methods.

[17] Kang S.-M., Lee J.-H., Huh Y.S., Takayama S. (2020). Alginate Microencapsulation for Three-Dimensional In Vitro Cell Culture. ACS Biomater. Sci. Eng..

[18] Rao W., Zhao S., Yu J., Lu X., Zynger D.L., He X. (2014). Enhanced enrichment of prostate cancer stem-like cells with miniaturized 3D culture in liquid core-hydrogel shell microcapsules. Biomaterials.

[19] Sakai S., Inamoto K., Ashida T., Takamura R., Taya M. (2015). Cancer stem cell marker-expressing cell-rich spheroid fabrication from PANC-1 cells using alginate microcapsules with spherical cavities templated by gelatin microparticles. Biotechnol. Prog..

[20] Stojkovska J., Zvicer J., Milivojevic M., Petrovic I., Stevanovic M., Obradovic B. (2020). Validation of a novel perfusion bioreactor system in cancer research. Hem. Ind..

[21] Hu C., Sun H., Liu Z., Chen Y., Chen Y., Wu H., Ren K. (2016). Freestanding 3-D microvascular networks made of alginate hydrogel as a universal tool to create microchannels inside hydrogels. Biomicrofluidics.

[22] Blaeser A., Duarte Campos D.F., Puster U., Richtering W., Stevens M.M., Fischer H. (2016). Controlling Shear Stress in 3D Bioprinting is a Key Factor to Balance Printing Resolution and Stem Cell Integrity. Adv. Healthc. Mater..

[23] Li Q., Lin H., Wang O., Qiu X., Kidambi S., Deleyrolle L.P., Reynolds B.A., Lei Y. (2016). Scalable Production of Glioblastoma Tumor-initiating Cells in 3 Dimension Thermoreversible Hydrogels. Sci. Rep..

[24] Li Q., Lin H., Rauch J., Deleyrolle L.P., Reynolds B.A., Viljoen H.J., Zhang C., Zhang C., Gu L., Van Wyk E. (2018). Scalable Culturing of Primary Human Glioblastoma Tumor-Initiating Cells with a Cell-Friendly Culture System. Sci. Rep..

[25] Chaicharoenaudomrung N., Kunhorm P., Promjantuek W., Heebkaew N., Rujanapun N., Noisa P. (2019). Fabrication of 3D calcium-alginate scaffolds for human glioblastoma modeling and anticancer drug response evaluation. J. Cell. Physiol..

[26] Ma L., Zhang B., Zhou C., Li Y., Li B., Yu M., Luo Y., Gao L., Zhang D., Xue Q. (2018). The comparison genomics analysis with glioblastoma multiforme (GBM) cells under 3D and 2D cell culture conditions. Colloids Surf. B Biointerfaces.

[27] Lee S.Y. (2016). Temozolomide resistance in glioblastoma multiforme. Genes Dis..

[28] Shojaei S., Koleini N., Samiei E., Aghaei M., Cole L.K., Alizadeh J., Islam M.I., Vosoughi A.-R., Albokashy M., Butterfield Y. (2020). Simvastatin increases temozolomide-induced cell death by targeting the fusion of autophagosomes and lysosomes. FEBS J..

[29] Ayuso J.M., Virumbrales-Munoz M., Lacueva A., Lanuza P.M., Checa-Chavarria E., Botella P., Fernandez E., Doblare M., Allison S.J., Phillips R.M. (2016). Development and characterization of a microfluidic model of the tumour microenvironment. Sci. Rep..

[30] Wang K., Kievit F.M., Erickson A.E., Silber J.R., Ellenbogen R.G., Zhang M. (2016). Culture on 3D Chitosan-Hyaluronic Acid Scaffolds Enhances Stem Cell Marker Expression and Drug Resistance in Human Glioblastoma Cancer Stem Cells. Adv. Healthc. Mater..

[31] Calatozzolo C., Gelati M., Ciusani E., Sciacca F.L., Pollo B., Cajola L., Marras C., Silvani A., Vitellaro-Zuccarello L., Croci D. (2005). Expression of drug resistance proteins Pgp, MRP1, MRP3, MRP5 and GST-pi in human glioma. J. Neuro-Oncol..

[32] Drean A., Rosenberg S., Lejeune F.X., Goli L., Nadaradjane A.A., Guehennec J., Schmitt C., Verreault M., Bielle F., Mokhtari K. (2018). ATP binding cassette (ABC) transporters: Expression and clinical value in glioblastoma. J. Neuro-Oncol..

[33] De Trizio I., Errede M., d’Amati A., Girolamo F., Virgintino D. (2020). Expression of P-gp in Glioblastoma: What we can Learn from Brain Development. Curr. Pharm. Des..

[34] Florczyk S.J., Wang K., Jana S., Wood D.L., Sytsma S.K., Sham J., Kievit F.M., Zhang M. (2013). Porous chitosan-hyaluronic acid scaffolds as a mimic of glioblastoma microenvironment ECM. Biomaterials.

[35] Munoz J.L., Walker N.D., Scotto K.W., Rameshwar P. (2015). Temozolomide competes for P-glycoprotein and contributes to chemoresistance in glioblastoma cells. Cancer Lett..

[36] Ozturk M.S., Lee V.K., Zou H., Friedel R.H., Intes X., Dai G. (2020). High-resolution tomographic analysis of in vitro 3D glioblastoma tumor model under long-term drug treatment. Sci. Adv..

